# Birth Preparedness and Complication Readiness among Pregnant Women in Duguna Fango District, Wolayta Zone, Ethiopia

**DOI:** 10.1371/journal.pone.0137570

**Published:** 2015-09-17

**Authors:** Merihun Gebre, Abebe Gebremariam, Tsedach Alemu Abebe

**Affiliations:** 1 Duguna Fango District health office, Wolayta, Ethiopia; 2 College of Public Health and Medical Sciences, Jimma University, Jimma, Ethiopia; London School of Economics, UNITED KINGDOM

## Abstract

**Background:**

Birth Preparedness and Complication Readiness is a strategy to promote the timely use of skilled maternal and neonatal care, especially during childbirth, based on the theory that preparing for childbirth and being ready for complications reduces delays in obtaining this care.

**Objective:**

This study was conducted to assess birth preparedness and complication readiness and its associated factors among pregnant woman in Duguna Fango District in Wolayta Zone, South Ethiopia.

**Methods:**

A community based cross-sectional study was conducted in 2013, on a sample of 578 pregnant women. Data were collected using pre-tested and structured questionnaire. The collected data were analyzed by SPSS for windows version 16.0. The women were asked whether they followed the desired five steps while pregnant: identified a trained birth attendant, identified a health facility, arranged for transport, identified blood donor and saved money for emergency. Taking at least three steps was considered being well-prepared.

**Results:**

Among 578 pregnant women only one tenth (10.7%) of pregnant women identified skilled provider. Only 103 (18.1%) arranged transportation to health facility. Two hundred forty eight (43.6%) identified health facility for delivery and/or for obstetric emergencies. more than half (54.1%) of families saved money for incurred costs of delivery and emergency if needed. only few 17(3%) identified potential blood donor in case of emergency. Two hundred sixty four (46.4%) of the respondents reported that they intended to deliver at home, and more than half (53.6) planned to deliver at health facilities. Overall less than one fifth 18.3% of pregnant women were well prepared. The adjusted multivariate model showed that significant predictors for being well-prepared were maternal availing of antenatal services (AOR = 2.95, 95% CI: 1.62–5.37), being pregnant for the first time (AOR = 3.37, 95% CI: 1.45–7.82), having knowledge of at least two danger signs during pregnancy (AOR = 2.81, 95% CI: 1.69–4.67) and history of past obstetric complication (AOR = 2.98, 95% CI: 1.35–6.58).

**Conclusion:**

Birth preparedness practice in the study area was found to be low. Information, Education and Communication (IEC) on birth preparedness and complication readiness for young people should start early adolescence. The government officials and partners that are working in areas of maternal health should come up with strategies to improve birth preparedness at individual and community level.

## Introduction

Maternal mortality remains a main challenge worldwide. According to assessment of maternal mortality in 180 countries, in 2010, it was estimated that 287,900 maternal deaths to occur annually. Developing country account about 99% (284,000) of global maternal death and more than half (162,000) of all maternal deaths were in the Sub Saharan Africa [1].

A woman’s lifetime risk of dying from pregnancy-related complications or during childbirth is one in 48 in the less developed world, versus only one in 1,800 in the developed world. The risk of dying from pregnancy-related causes is highest in Africa, because as some literatures indicate African women give more children than women on other continents and also because the risks are greater with each pregnancy. Complications from pregnancy and childbirth are the leading causes of death among women of reproductive age in India and worldwide [2, 3].

Maternal deaths have both direct and indirect causes. About 80 percent of maternal deaths are due to causes directly related to pregnancy and childbirth—unsafe abortion and obstetric complications such as severe bleeding, infection, hypertensive disorders, and obstructed labor among those pregnant who do not have visited health care service or inadequate care [2].

According to EDHS 2012 report, in Ethiopia, the maternal mortality rate is high which is 676 per 100,000 live births which remains a major public health challenge [4].

Many countries, particularly in sub-Saharan Africa and South/Southeast Asia, still have unsatisfactory levels of the recommended four or more antenatal care visits [5].

Only ten percent of births in Ethiopia are delivered at a health facility—9 percent in a public facility and 1 percent in a private facility. Nine women in every ten deliver at home. By comparison, in 2005 28 percent received antenatal care from a skilled provider [4].

Women and newborns need timely access to skilled care during pregnancy, childbirth, and the postpartum/newborn period. Too often, however, their access to care is impeded by delays—delays in deciding to seek care, delays in reaching care, and delays in receiving care. These delays have many causes, including logistical and financial concerns, unsupportive policies, and gaps in services, as well as inadequate community and family awareness and knowledge about maternal and newborn health issues. The attitudes and behaviors of skilled providers toward clients were described by respondents as a major barrier to use of skilled care [6, 7].

Pregnant women who do not have adequate and appropriate information about pregnancy and childbirth would be ill-equipped to make choices that will contribute to their own well-being [8].

Antenatal care should place emphasis on birth preparedness and complication readiness to improve access to skilled and emergency obstetric care which has been shown to be critical in reducing maternal and/or perinatal mortality and morbidity [9].

Since it is not possible to predict which women will experience life-threatening obstetric complications that lead to maternal mortality, every pregnant woman faces the risk of sudden, unpredictable complications that could end in death or injury to herself or her infant. So receiving care from a skilled provider (doctor, nurse or midwife) during childbirth has been identified as the single most important intervention in safe motherhood [4].

Birth Preparedness and Complication Readiness (BPACR) is a strategy to promote the timely use of skilled maternal and neonatal care, especially during childbirth, based on the theory that preparing for childbirth and being ready for complications reduces delays in obtaining this care. It entails making plans prior to birth to ensure that a pregnant woman is prepared for normal birth and complications. It can positively influence knowledge and intermediate health outcomes, such as household practices and use of some health services [8, 10]. Despite the fact that birth preparedness and complication readiness is essential for further improvement of maternal and child health little is known about the current magnitude and influencing factors in Ethiopia. This study therefore aims to fill this gap by assessing the current status and factors associated with birth preparedness and complication readiness among pregnant women in Duguna Fango District in Wolayta Zone, South Ethiopia, through a community based cross sectional study. It is hoped that the results of the study will provide valuable information for design of possible programs and interventions to improve maternal and neonatal health. And also serve as baseline information for further study.

## Methods and Materials

### Study area

The study was conducted in Duguna Fango District in Wolayta Zone, which is located 362 kilometers south of Addis Ababa. Administratively the district is divided in to 26 rural and 2 urban sub-cities (Kebeles). As projected from Central Stastical Agency (CSA) 2007 report, the district has a total of 114,359 (56,290 males and 58,069 females) living in 23,338 households. An estimated 4,460 were pregnant during 2013 [11, 12]. There are 6 health centers and 29 health posts in the district.

### Study design

In September 2013 a community based cross sectional study was conducted using quantitative methods. The study was conducted among all pregnant women who were residing in Duguna Fango District during the study period. The inclusion criteria were Women, with at least 3 months of current pregnancy, permanent resident of the study area, volunteer to participate and respond to the questionnaire were included. Women who were mentally disabled and severely ill were excluded.

### Sample size and sampling technique

The sample size calculation was determined by using a single population proportion formula for cross sectional study, based on the following assumptions pregnant women in the District were estimated to be about 3.9% of 114,359 = 4460 pregnant women. Prevalence of Birth preparedness and complication readiness was 22% [13]. The margin of error and confidence interval were taken to be 5% and 95% respectively. Considering the design effect of 2 and 10% non-response rate, the total sample size became 578,

$$\begin{array}{l} \text{n}={\left({\text{Z}}_{\text{a}/2}\right)}^{2}\;\text{p}\;\left(1\text{-p}\right)/{\text{d}}^{2}\\ \text{n}={\left(1.96\right)}^{2}\;0.22\left(0.78\right)/\;{\left(0.05\right)}^{2}=263\\ \text{n}=263+10\%=289\\ \text{n}=289*2=578 \end{array}$$

Multistage sampling procedure was used to select study subjects. First, all the kebeles in the District were stratified in to urban and rural. Then 1 urban and 8 rural kebeles were randomly selected for the study. The calculated sample size was proportionally allocated to urban and rural according to their population. Then a census was conducted to register all pregnant women and their gestational age. Based on the above information a sampling list, which enlists all eligible study subjects, was prepared. From the list, pregnant women with gestational age of 3 months and above were included in the survey. Using sampling frame generated from the census conducted, mothers in each keble were selected by computer generated random method.

### Measurement

A pre tested Structured interview questionnaire was used for data collection. It was taken from the monitoring birth preparedness and complication readiness: tools and Indicators for maternal and newborn health Jhpiego, an affiliate of John Hopkins University [8] and adapted according to local context and the objectives of the study. Using a pre tested questionnaire the following information were collected. Socio demographic characteristics including: age, marital status, residence (urban versus rural), ethnicity, religion, education, occupation and average monthly family income. The questionnaire included questions health problems during previous pregnancies, Danger signs during pregnancy, delivery and postnatal period which require referral and whether the mother follows the following basic five steps were asked i) identified a trained birth attendant or ii) health facility for emergency; iii) identified mode of transport for delivery and/or for obstetric emergency; iv) saved money and v) identified blood donor. The women were asked about antenatal care services and number of visits, who attended the ANC, preferred place of delivery.

### Data collection process

Six experienced female data collectors were collected the data after thorough training on the objective of the study and the questionnaire. Two degree holder health professionals supervised the data collectors. Data collectors and supervisors were trained for 4 days by using training manual prepared for this purpose.

### Data analysis

The collected data were coded, entered, and cleaned, and analyzed by SPSS for windows version 16.0. First, simple frequency distribution was calculated. Then those mothers who followed at least three of the five Birth Preparedness and Complication Readiness were considered ‘‘well prepared”. The remaining pregnant women were considered ‘‘less prepared”. Logistic regression analysis was done to identify factors associated with Birth Preparedness and Complication Readiness.

### Data quality control

To maintain the validity of the measurement standard questionnaire of monitoring birth preparedness and complication readiness: tools and Indicators for maternal and newborn health was taken and modified based on study interest. The instrument was pre tested on 5% of sample size in Damote Woyde district that was not included in study and analysis. Modifications were made after pretest. The questions were translated to local language (Wolayta language and Amharic) and back translated to English to maintain consistency. The translators were well known translators for both languages. Training was given to data collectors and supervisors. Observation and supervision was done throughout the fieldwork, training and data collection process. In addition meeting with each member of the team on a daily basis to discuss performance and give out future work assignments was performed.

### Ethical considerations

Ethical approval was obtained from Ethical Review Committee of Jimma University. Letter of support was obtained from the Zonal and District health offices before undertaking the study and written informed consent was obtained from the respondents before the interview. For Privacy and confidentiality, all interviews were conducted in private and all cautions were taken to ensure confidentiality. The right of the respondents to refuse to participate in the study was respected. Respondents were provided information on importance of antenatal care and birth preparedness and complication readiness.

## Results

### Socio-demographic and obstetric characteristics

A total of 578 pregnant women were identified to participate in the study. Out of these 569 were interviewed making a response rate of 98.4%. The rest were not found for an interview after three repeated visits. The mean ± Standard Deviation age of respondents was 26 ± 4 years. Of the respondents 560 (98.4%) were currently in marital union. Large Majority 538(94.8%) were rural dwellers. By ethnicity 547(96.1%) were Wolaytas and 20(3.5%) were Amhara. The major predominant religions include protestant 402(70.7%) and Orthodox Christians 146(25.7%). Educationally 398 (69.9%) of respondents had no formal education. Occupationally 524(92.1%) were housewives followed by government employees 28 (4.9%). The majority of respondents^’^ family income 381(67.8%) were less than 500 Ethiopia Birr. Regarding pregnancy status majority 365(64.1%) of the respondents had already given birth for two to four children; 106(18.7%) had delivered five and more and the remainders 98(17.2%) were primigravidae. (Table 1).

**Table 1:** Socio-demographic and obstetric characteristics of pregnant women in Duguna Fango District, 2013 (n = 569). This table shows the socio-demographic and obstetric characteristics of the participants in the study area.

Characteristic	Number(n)	Percent (%)
**Residence**		
Urban	31	5.4
Rural	538	94.6
**Age**		
<20	55	9.7
20–25	240	42.2
26–30	213	37.4
>30	61	10.7
**Marital status**		
Married	560	98.4
Not married	9	1.6
**Family income**		
<500	381	67
500–1000	153	26.9
>1000	35	6.1
**Ethnicity**		
Wolayta	547	96.1
Amhara	20	3.5
Hadiya	2	0.4
**Religion**		
Protestant	402	70.6
Orthodox	146	25.7
Catholic	21	3.7
**Occupation**		
House wife	524	92.1
Government employee	28	4.9
Private employee	3	0.5
Others	14	2.5
**Maternal education**		
Illiterate	272	47.8
Read and write	128	22.5
primary	118	20.7
Secondary and above	51	9
**Husband occupation(n = 560)**		
Farmer	454	81.1
Government employee	37	6.6
Private employee	25	4.5
Merchant	44	7.8
**Husbands education**		
Illiterate	169	30.2
Read and write	129	23
Primary	152	27.1
Secondary and above	110	19.7
**Parity**		
Para 0	98	17.2
Para 2–4	365	64.1
Para ≥ 5	106	18.7

***Others*:** *farmer*, *student*, *self employee*, *and jobless*

### Antenatal care and advice given during current pregnancy

About 342 (60.1%) of respondents attended antenatal care in their current pregnancy. The mean antenatal attendance was 2 + 0.7. std. The respondents’ mean gestational age at first antenatal visit was 4.7 months. Majority 251 (73%) of respondents reported a Health care provider had given health advice during their ANC visit. About 271 (79.2%) of respondents were given advice where to go if health problems happen; Three hundred twelve (91.2%) of respondents were given advice where to deliver, 96 (28.1%) were advised to arrange transportation to go health facility, 150 (43.8%) were advised to save money for expense of emergency and birth related conditions, only few 20 (5.8%) were advised to arrange compatibly blood donor for emergency and 138 (40.8%) were advised to identify skilled birth attendant (Table 2).

**Table 2:** Antenatal care services and advice given among pregnant women in Duguna Fango District, 2013 (n = 569). This table shows antenatal care services and advice given among pregnant women.

Characteristics	Number(n)	Percent (%)
**Antenatal care attended during**		
**current pregnancy (n = 569)**		
Yes	342	60.1
No	227	39.9
**Gestational age at first antenatal visit (n = 342)**		
1^st^ trimester	45	13.1
2^nd^ trimester	278	81.3
3^rd^ trimester	19	5.6
**Personnel checked (n = 342)**		
Health professional	251	73.0
Health extension worker	91	27.0
**Advise on danger signs(n = 342)**		
Yes	285	83.3
No	57	16.7
**Advise on where to go if danger signs happen(n = 342)**		
Yes	271	79.2
No	71	20.8
**Advise on identifying health facility(n = 342)**		
Yes	312	91.2
No	30	8.8
**Advise on arrangement for transport (n = 342)**		
Yes	96	28.1
No	246	71.9
**Advise on saving money for delivery**		
**or emergency (n = 342)**		
Yes	150	43.9
No	192	56.1
**Advise on arranging blood donor**		
**in case of emergency (n = 342)**		
Yes	20	5.8
No	322	94.2
**Advise on identifying skilled birth**		
**attendant (n = 342)**		
Yes	139	40.6
No	203	59.4

**Fig 1:**
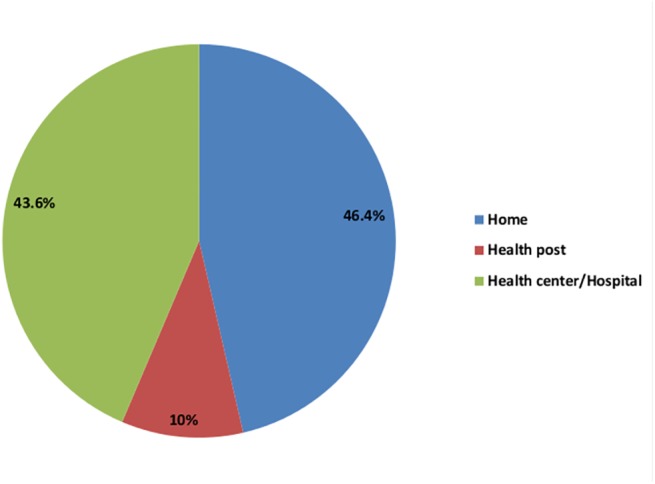
Expected place of delivery among pregnant women in Duguna Fango District, 2013.This figure shows planned for place of delivery among pregnant women in Duguna Fango District. Two hundred sixty four (46.4%) of pregnant women planned to give birth at home, 248 (43.6%) pregnant women planned health center/ hospital birth and 57 (10%) of respondents planned health post for child birth.

Two hundred sixty four (46.4%) of pregnant women planned to give birth at home, 248 (43.6%) pregnant women planned health center/ hospital birth and 57 (10%) of respondents planned health post for child birth (Fig 1).

### Birth preparedness practices

Only one tenth (10.7%) of pregnant women identified skilled provider. Two hundred forty eight (43.6%) identified health facility for delivery and/or for obstetric emergencies. More than half (54.1%) of families saved money for incurred costs of delivery and emergency if needed. About 103 (18.1%) arranged transportation to health facility. Preparedness for compatible blood donor was found to be very low (3%) (Table 3). The birth preparedness score was computed from key elements of birth preparedness such as; arrangement for transportation, saving money for delivery, identified skilled attendant to assist at birth, identifying a health facility for emergency and identifying blood donor in case of emergency. Taking at least three steps was considered being well prepared. Accordingly less than one fifth 18.3% of pregnant women on this study were considered as well prepared for birth and complications.

**Table 3:** Birth preparedness and complication readiness among pregnant women in Duguna Fango District, 2013 (n = 569). This table shows Birth preparedness and complication readiness among pregnant women.

Level of birth preparedness and complication readiness	Number(n)	Percent (%)
**Identified health facility**		
Yes	248	43.6
No	321	56.4
**Arranged transport**		
Yes	103	18.1
No	466	81.9
**Save money**		
Yes	308	54.1
No	261	45.9
**Identified compatible blood donor**		
Yes	17	3.0
No	552	97.0
**Identified skilled birth attendant**		
Yes	62	10.9
No	507	89.1
**Number of steps taken**		
0	202	35.5
1	144	25.3
2	119	20.9
3	66	11.6
4	26	4.6
5	12	2.1
At least three steps taken	104	18.3

### Factors associated with birth preparedness and complication readiness

The adjusted multivariate model showed that significant predictors for being well-prepared maternal availing of antenatal services (AOR = 2.95, 95% CI: 1.62–5.37), being pregnant for the first time (AOR = 3.37, 95% CI: 1.45–7.82), history of past obstetric complications (AOR = 2.98, 95% CI: 1.35–6.58) and having knowledge of at least two danger signs during pregnancy (AOR = 2.81, 95% CI: 1.69–4.67) (Table 4).

**Table 4:** Association of selected variables with Birth preparedness and complication readiness, Duguna Fango District, 2013 (n = 569). This table shows the factors associated with Birth preparedness and complication readiness.

Variables	Birth preparedness		Crude OR	Adjusted OR
	Number(percent)		OR(95% CI)	OR(95% CI)
	Well prepared(n = 104)	Less prepared(n = 465)		
**Age**				
≤ 25 year	61(58.7)	234(50.3)	1.40(0.91–2.15)	0.91(0.52–1.59)
> 25 year	43(41.3)	231(49.7)	1.0	1.0
**Residence**				
Urban	13(12.5)	20(4.3)	3.17(1.52–6.62)	1.76(0.75–4.14)
Rural	91(87.5)	445(95.7)	1.0	1.0
**Parity**				
0	27(26.0)	71(15.3)	2.06(1.10–4.17)	3.37(1.45–7.82)*
2–4	65(62.5)	300(64.5)	1.26(0.69–2.28)	1.84(0.89–3.79)
≥5	12(11.5)	94(20.2)	1.0	1.0
**Monthly income**				
≤ 500birr	62(59.6)	319(68.6)	1.0	1.0
>500birr	42(40.4)	146(31.4)	1.48(0.95–2.29)	1.37(0.83–2.26)
**Maternal education**				
No formal education	66(63.5)	332(71.4)	1.0	1.0
Formal education	38(36.5)	133(28.6)	0.69(0.45–1.08)	0.68(0.39–1.17)
**Husband education**				
No formal education	49(47.1)	249(53.5)	1.0	1.0
Formal education	55(52.9)	216(46.5)	1.29(0.84–1.98)	0.83(0.48–1.42)
**Knowledge of at least two danger signs during pregnancy**				
Yes	73(70.2)	157(33.8)	4.62(2.91–6.33)	2.81(1.69–4.67)**
No	31(29.8)	308(66.2)	1.0	1.0
**Knowledge of at least two danger signs during childbirth**				
Yes	90(86.5)	293(63.0)	3.77(2.08–5.83)	1.72(0.87–3.37)
No	14(13.5)	172(37.0)	1.0	1.0
**Knowledge of at least two danger signs during postpartum**				
Yes	58(55.8)	106(22.8)	4.27(2.74–6.65)	3.09(1.88–5.10)**
No	46(44.2)	359(77.2)	1.0	1.0
**ANC attendance**				
Yes	87(83.7)	255(54.8)	4.21(2.42–7.31)	2.95(1.62–5.37)**
No	17(16.3)	210(45.2)	1.0	1.0
**History of complication**				
Yes	17(16.3)	21(4.5)	4.13(2.09–7.81)	2.98(1.35–6.58)*
No	87(83.7)	444(95.5)	1.0	1.0

* Variables significant at p< 0.05

** Variables significant at p< 0.01

## Discussion

In this study several important findings were observed. Availing antenatal care, being pregnant for the first time, history of past obstetric complications and having knowledge of at least two danger signs during pregnancy were predictors of birth preparedness and complication readiness.

The study showed that however only 18.3% of pregnant women were well prepared for birth and obstetric emergency by considering preparation. This finding is lower than a study done in India 47.8% [14] and Uganda 35% [15]. This could be due to the fact that the difference in study area /socio-cultural characteristics/ and implementation of related health program.

Another important finding in this study was women who attended antenatal care service were well prepared than those who did not attend. The finding is similar to other studies conducted in Adigrate Ethiopia, India and Aleta Wondo [13, 14, 16]. This signifies that antenatal care services visits are an opportunity to inform pregnant women and help to plan for the important components of birth preparedness and complication readiness. In our study women with first pregnancy were more prepared than their counterparts. This could be high risk perception of such women than those who had experience. This shows that increasing risk perception might help in improving BPACR.

The study found that women who have knowledge of at least two key danger signs during pregnancy were more likely to be well prepared, which is consistent with study done in Uganda [15]. This is could be knowledge of danger signs of obstetric complications is essential for women to seek skilled birth attendants. The study revealed that women with history of obstetric complication were more likely to be well prepared than their counterparts. This finding is similar with a study done in Adigrat, Ethiopia [13]. This could be the reason that women perceive serious problems based on previous experiences.

In case of an obstetric emergency arrangement of transportation to health facility is important. However, Only 18.1% of pregnant women arranged transportation. This is less than findings from Adigrat 24.7% and India 29.5% [13, 14]. This could be differences in the local contexts. In our setup the community uses traditional ways such as donkey cart and local stretchers to carry patients to facilities. Unavailability of roads in some of the rural setups plays role. Therefore messages on BPACR should be tailor to the local contexts and doe able messages.

Another finding of this study was only 10.9% of women identified a skilled birth attendant. While increasing knowledge to prepare for birth and emergencies is important, efforts are required to identify barriers for use skilled attendants at birth. In this study Identified compatible blood donor was found to be very low (3%). This finding indicated that women were not informed well all the components of BPACR. This implies the importance of training for health providers on how to advise pregnant women on components of BPACR.

The strength of the study includes it is a community based; census was conducted before data collection to identify currently pregnant women. The limitations of the study are: since the participants have not completed their pregnancies, they may not yet have had the opportunity or need to make arrangements related to BPACR. Pregnant women may not able to report whether they used services that they have not yet needed.

This study revealed that only few of pregnant women were well prepared for delivery and obstetric complication, a large majority of pregnant women planned to deliver at home where the presence of skilled attendants is uncertain. In addition only small percentage of pregnant women Identified blood donor and transportation.

Our study showed that availing antenatal care services, being pregnant for the first time, history of past obstetric complications and having knowledge of at least two danger signs during pregnancy were predictors of BPACR.

Therefore Information, Education and Communication (IEC) on birth preparedness and complication readiness for young people should start early adolescence. The government officials and partners that are working in areas of maternal health should come up with strategies to improve birth preparedness at individual and community level.
